# Multimodal Imaging Reveals Cerebral Microcirculation Dynamics and Mechanisms in Mouse Central Nervous System After Intranasal Infection With Recombinant Pseudorabies Virus

**DOI:** 10.1002/advs.202508124

**Published:** 2025-09-23

**Authors:** Shuting Ling, Chongxin Wu, Mengxuan Gui, Kaiyun Chen, Yanbo Yang, Jiwei Xing, Fengxian Du, Wei Liao, Luyao Yang, Zhaokui Jin, Ningshao Xia, Guosong Wang, Yixin Chen, Qingliang Zhao

**Affiliations:** ^1^ State Key Laboratory of Vaccines for Infectious Diseases Xiang An Biomedicine Laboratory National Innovation Platform for Industry‐Education Integration in Vaccine Research Center for Molecular Imaging and Translational Medicine School of Public Health Xiamen University Xiamen Fujian 361102 China; ^2^ State Key Laboratory of Molecular Vaccinology and Molecular Diagnostics National Institute of Diagnostics and Vaccine Development in Infectious Diseases School of Life Sciences School of Public Health Xiamen University Fujian Province Xiamen 361102 China; ^3^ School of Biomedical Engineering Guangzhou Medical University Guangzhou 511436 China; ^4^ Department of Experimental Research Sichuan Clinical Research Center for Cancer Sichuan Cancer Hospital & Institute Sichuan Cancer Center University of Electronic Science and Technology of China Chengdu 610042 China

**Keywords:** central nervous system, cerebral imaging, multiscale imaging, pathogenic mechanism, pseudorabies virus

## Abstract

Neurotropic viruses are highly invasive in the central nervous system (CNS) and can disrupt the microcirculation, leading to neurological complications and encephalopathy. Understanding these pathophysiological alterations is crucial for uncovering the underlying mechanisms of viral infection, developing effective vaccines, and creating targeted therapies. However, studying the cerebral microcirculation in vivo during infection is challenging. This study engineered the pseudorabies virus (PRV) Fa‐Luc using the EP0‐sgRNA‐hSPCas9 system to investigate cerebral microcirculation dynamics during PRV‐induced CNS infection. Moreover, a multi‐modality imaging strategy that integrates macroscale magnetic resonance imaging, bioluminescence with microscale laser speckles, optical coherence tomography angiography, and two‐photon imaging for multiscale analysis is developed. The results revealed an initial hyperemic response in the dorsal cortical blood flow, which later declined, especially in the primary visual cortex, along with notable vascular structural alterations, including abnormal dilation, in the later stages of infection. Furthermore, transcriptome sequencing suggested that PRV infection triggers immune activation and the release of inflammatory factors, leading to endothelial dysfunction and the inhibition of vascular development. This study offers novel insights into the pathogenesis of neurotropic viruses and provides a valuable tool for research on CNS infections and vaccine development.

## Graphical abstract

Multi‐modality imaging strategy applied to PRV‐induced central nervous system infection enables dynamic visualization of cerebrovascular dysfunction, including hyperemia, hypoperfusion, and vascular remodeling. These changes are linked to microglial activation and impaired angiogenesis, providing mechanistic insight into PRV neuropathogenesis and supporting research on CNS infection and therapeutic development.

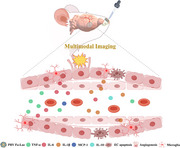

## 1 Introduction

The coronavirus disease (COVID‐19) pandemic emerged in 2019 and rapidly spread globally, resulting in ≈7 million deaths^[^ ^1^, ^2^, ^3^ ^]^ Viral infections disrupt central nervous system (CNS) function, increasing vascular inflammation and cerebral thrombosis in conditions such as stroke. This indicates that endothelial inflammation and blood hypercoagulability are responsible for these events.^[^ ^4^, ^5^, ^6^, ^7^ ^]^ Given the complexity of immune mechanisms and barrier protection in the CNS, viral infections frequently encounter substantial challenges in peripheral tissue.^[^ ^8^, ^9^ ^]^ However, some viruses, such as polioviruses and alpha‐herpesviruses (α‐HVs), can circumvent these defensive mechanisms and invade the CNS.^[^ ^10^, ^11^, ^12^ ^]^ Pseudorabies virus (PRV), a member of the α‐HV subfamily known as porcine herpesvirus type 1, can retrogradely disseminate through the trigeminal ganglion upon entering the nerve endings of the peripheral nervous system, eventually invading the CNS. Furthermore, PRV particles can migrate to the ganglion where they remain latent, posing a long‐term risk to host health.^[^ ^13^, ^14^, ^15^ ^]^ This highlights the importance of PRV as a valuable viral model for studying the molecular biology, pathogenesis, neuroinvasion, and transneuronal spread of herpesviruses.^[^ ^16^ ^‐^ ^19^ ^]^

In addition, cerebral microcirculation plays a pivotal role in maintaining CNS homeostasis, supplying nutrients and oxygen to neurons, and facilitating the removal of waste.^[^ ^20^, ^21^ ^]^ As a dynamic and complex network, the cerebral microcirculatory system consists of capillaries, arterioles, and venules that interact to regulate blood flow in response to various physiological demands. This microvascular network is essential in the context of neurotropic viral infections, which can disrupt normal brain function and lead to severe neurological consequences.^[^ ^22^, ^23^, ^24^ ^]^ Furthermore, coordinated regulation of cerebral blood flow and lymphatic circulation facilitates efficient viral clearance from the nervous system.^[^ ^25^, ^26^, ^27^, ^28^ ^]^ Thus, the intricate interplay between cerebral microcirculation and neurotropic viral infections presents unique insights and significant challenges in the study of neurovascular pathologies.

Various imaging techniques have been employed to study cerebral blood flow and vascular integrity in vivo, investigating the interaction between cerebral microcirculation and neurotropic viral infections. Despite advancements in imaging technologies, such as magnetic resonance imaging (MRI) and molecular techniques, including positron emission tomography, the interaction of neurotropic viruses with cerebral microcirculation during infection remains unclear.^[^ ^29^, ^30^, ^31^, ^32^ ^]^ One primary challenge is the complexity of the brain‐blood barrier, which poses difficulties in simulating the natural environment of neurotropic viral invasions. Additionally, because of the limitations of a single imaging modality in terms of spatial and temporal resolution, imaging depth, physiological parameter acquisition, and biocompatibility, a multimodal imaging approach is often required to investigate complex biomedical problems.^[^ ^33^, ^34^ ^]^ Moreover, the ability to monitor cerebral microcirculation in real‐time during infection could help determine the underlying mechanisms and lead to the development of vaccines for early precautions and targeted therapies. Hence, understanding the interplay between the cerebral microcirculation and neurotropic viral infections is crucial for developing novel therapeutic strategies aimed at preventing viral invasion and improving viral clearance within the CNS.

To address these challenges, a recombinant virus, PRV Fa‐Luc, was constructed using the EP0‐sgRNA‐hSPCas9 gene‐editing system, and an intranasal infection pathway that reliably mimicked the essential characteristics of human CNS infection was validated through living tracing. Neurotropic viral infections can disrupt the cerebral microcirculation by altering vascular structure, impairing blood flow regulation, and compromising endothelial function. A single modality may fail to comprehensively assess these interconnected factors. Thus, a multimodal imaging platform was developed that combines macroscale MRI and bioluminescence with microscale laser speckles, optical coherence tomography angiography (OCTA), and two‐photon imaging for multiscale investigation. Moreover, multimodal cerebrovascular imaging revealed an initial hyperemic response in the dorsal cortical blood flow, followed by a decline, particularly in the primary visual cortex, accompanied by significant vascular structural changes, including abnormal dilation, at later stages of infection. Transcriptomic sequencing analysis was performed to further investigate the effect of PRV CNS infection on gene expression in brain tissue. These results suggest that neurotropic viral infection may hinder normal blood vessel development, reinforcing the observations from multimodal imaging. These findings provide a crucial framework for understanding the interplay between viral neuroinvasion and cerebrovascular dynamics, offering novel insights into CNS infections and their broad effect on neural and vascular health. Ultimately, this study can contribute to improved diagnosis, management, and treatment of neurotropic viral infections, including emerging viral threats that may present unique challenges to the cerebrovascular system.

## 2 Results

### 2.1 Construction and Characterization of Recombinant Virus PRV Fa‐Luc

**Figure 1:**
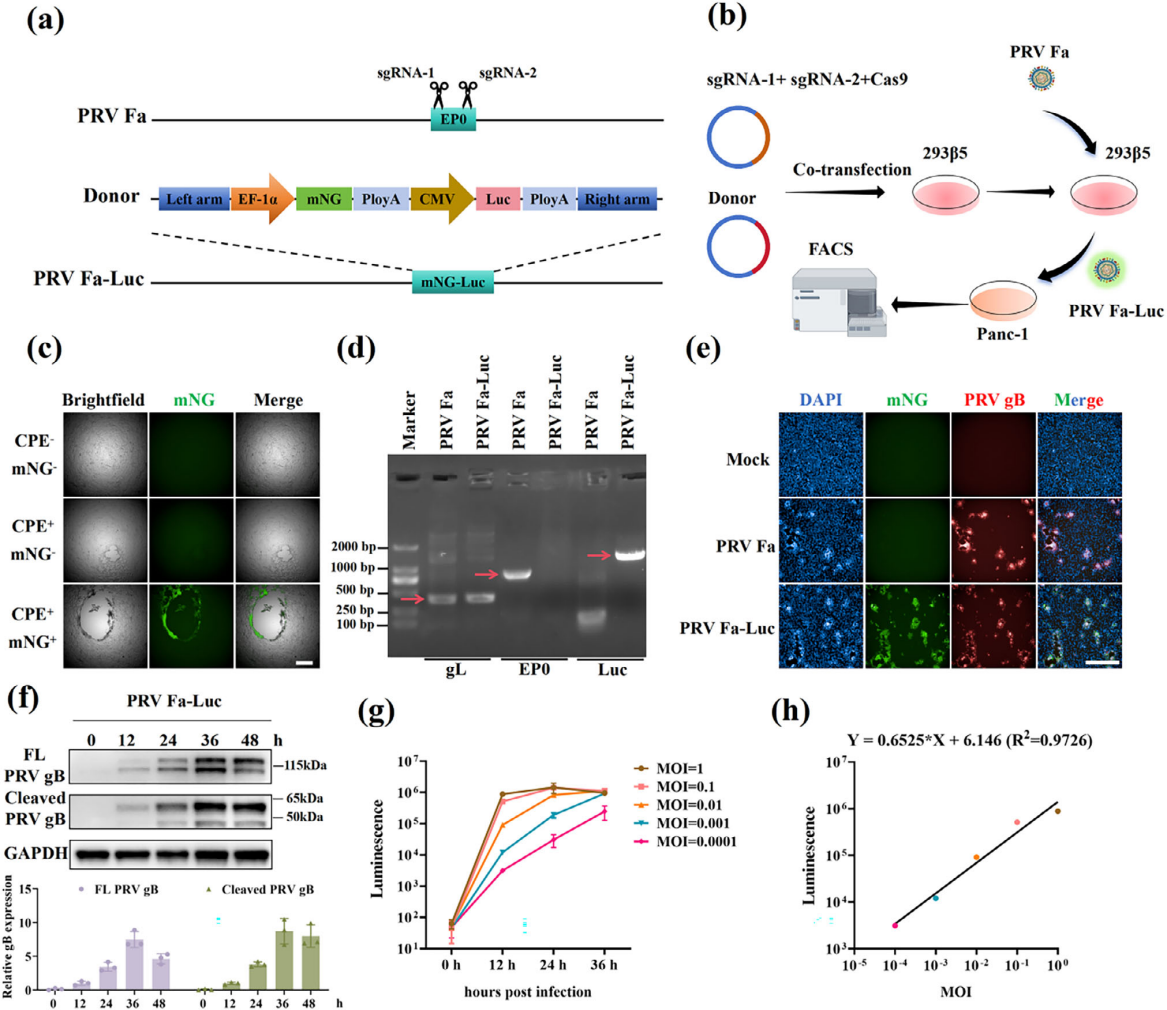
Construction and identification of recombinant PRV Fa‐Luc. a) Schematic diagram of the genome of PRV Fa‐Luc strain. b) Flowchart of the CRISPR/Cas9 experiment for obtaining the PRV Fa‐Luc strain. c) High‐content imaging systems are used for the rapid screening of wells in which cells infected by the PRV virus express mNG. CPE, cytopathic effect. d) Identification of the *EP0* gene knock‐out and *Luc* gene knock‐in in the genome of the PRV Fa‐Luc strain using PCR, with the *gL* gene serving as a positive control. e) Immunofluorescence assay to verify the expression of PRV gB in MDCK cells infected by the PRV‐Fa and PRV Fa‐Luc strains. Scale bar: 500 µm. f) The expression level of PRV gB in MDCK cells infected with the PRV Fa‐Luc was detected by Western blot at 0, 12‐, 24‐, 36‐, and 48‐h post‐infection. Quantitative analysis of protein bands using ImageJ. FL, full length (n = 3). g) The kinetic curve of luciferase activity in GBM cells following infection with PRV virus at different MOIs. h) Correlation analysis between luciferase activity in GBM cells 12 h post‐infection and MOIs with PRV Fa‐Luc. Data shown as mean ± SD.

The PRV early protein 0 (*EP0*) gene is located within the early transcriptional unit of the PRV genome. The EP0 protein, which is encoded by this gene, is one of the three proteins expressed early in PRV. It is expressed in the host within 2 h of infection.^[^ ^35^, ^36^, ^37^ ^]^ However, EP0 is not essential for viral transcription or replication.^[^ ^38^ ^]^ The CRISPR/Cas9 system was used to delete the *EP0* gene and insert the *mNeonGreen (mNG)* gene, along with a promoter and the *firefly luciferase* gene, into the PRV Fa strain genome (**Figure** **1**a). The gene‐edited supernatant was then collected and used to infect Panc‐1 cells. Single‐cell sorting was performed using flow cytometry to isolate green fluorescent cells from a 96‐well plate pre‐seeded with GBM cells (Figure 1b). High‐content screening identified the wells with significant mNG signals (Figure 1c). PCR analysis confirmed that the virus genome had the *EP0* gene deleted and the *Luc* gene was inserted (Figure 1d). This modified virus exhibited robust infectivity akin to that of the PRV Fa strain and demonstrated high PRV gB protein expression, with levels increasing over time (Figure 1e,f). Notably, the gB band at 24 h post‐infection displayed an uneven pattern due to the presence of both full‐length gB and proteolytically cleaved fragments, consistent with known gB processing mechanisms.^[^ ^39^, ^40^ ^]^ Cells infected with varying doses of PRV Fa‐Luc showed an increase in luciferase activity over time, confirming the efficient expression of Luc in PRV Fa‐Luc‐infected cells (Figure 1g). Additionally, at 12 h post‐infection, a significant positive correlation was observed between viral dose and luciferase activity (R^2^ = 0.9726) (Figure 1h). This finding indicates that luciferase activity can, to a certain extent, serve as a marker of the viral load.

### 2.2 Biological Characterization of Recombinant Virus PRV Fa‐Luc

**Figure 2:**
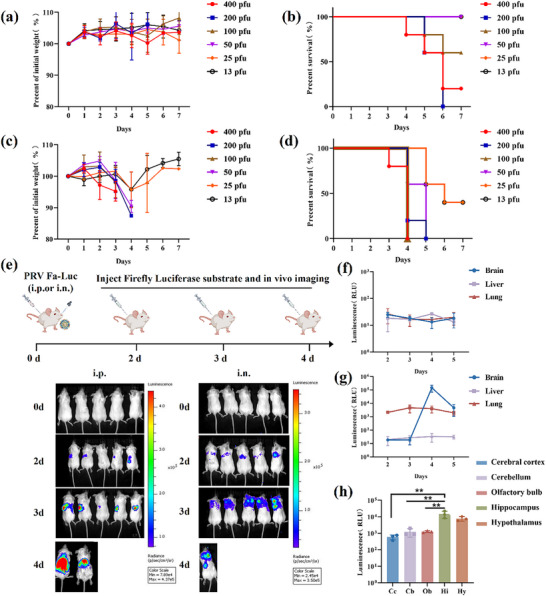
In vivo tracing of recombinant virus. a, b) Weight curve a) and survival curve b) of mice infected with various pfu of PRV Fa‐Luc via intraperitoneal injection (n = 5). c,d) Weight curve c) and survival curve d) of mice infected with various pfu of PRV Fa‐Luc via intranasal injection (n = 5). e) Distribution image of PRV Fa‐Luc in a mouse model (n = 5). f) The luciferase activity in liver, lung, and brain tissues of mice at days 2, 3, 4, 5 post intraperitoneal infection of PRV Fa‐Luc (n = 5). g) The luciferase activity in the liver, lung, and brain tissues of mice infected with PRV Fa‐Luc at days 2, 3, 4, 5 post intranasal infection of PRV Fa‐Luc (n = 5). h) The luciferase activity in brain regions of mice at day 4 post intranasal infection of PRV Fa‐Luc (n = 3). Data shown as mean ± SD. One‐way ANOVA in (g) followed by Bonferroni *t*‐test. **p < 0.01.

The pathogenicity of PRV Fa‐Luc was investigated by observing and documenting the clinical symptoms and survival rates of mice infected with the virus. Mice in the intraperitoneal infection group exhibited milder clinical symptoms, with mice in each dose group surviving for an average of 4–6 days (**Figure** **2**a,b). In the intranasal infection group, mice began to exhibit clinical symptoms, including hunched posture, anorexia, and severe pruritus on the head, 3 days post‐infection (dpi). The body weights of mice in the high (400 and 200 pfu) and medium (100 and 50 pfu) dose groups decreased significantly at 2 dpi, and the mice survived for an average of 4–5 days. In the low‐dose groups (25 and 13 pfu), the survival rate was up to 40%, and the body weight recovered between 4 and 7 dpi (Figure 2c,d). Based on these observations, 400 pfu was selected for intraperitoneal infection and 100 pfu for intranasal infection for subsequent experiments. This strategy aims to achieve a balance between robust clinical manifestations and consistent mortality, which is essential for establishing a reliable infection model. Similar approaches have been recommended for other viral infection models, in which suprathreshold doses are commonly used to ensure reproducible pathogenesis without excessive variability in the host response.^[^ ^41^, ^42^ ^]^ Notably, despite the difference in viral loads, both dosing regimens yielded comparable disease outcomes, suggesting that the route of infection may have a more substantial effect on disease severity than the absolute dose. Accordingly, 2, 3, and 4 dpi were focused upon, as preliminary data indicated that clinical symptoms and viral replication were not apparent at 1 dpi, but began to manifest from 2 dpi onward. This timeframe allowed the identification of the essential phases of disease progression and pathological changes.

### 2.3 In vivo Tracing of Recombinant Virus PRV Fa‐Luc

In vivo bioluminescence imaging was performed to track the recombinant virus and explore the potential variations in the distribution of PRV within mice under different infection routes. Significant viral signals were detected in the mouse model at 2 dpi. In the intraperitoneal group, bioluminescent signals were detected primarily in the abdomen, whereas in the intranasal group, the viral signals were mainly distributed in the chest at 2 dpi and in the brain during the later period; the signals gradually increased over time (Figure 2e). The firefly luciferase activity analysis showed similar levels in the liver, lungs, and brain tissues after intraperitoneal infection, and the replication of the virus was not apparent over time (Figure 2f). This phenomenon may be explained by the fact that intraperitoneally delivered viruses tend to remain confined to the peritoneal cavity and associated tissues, such as the peritoneum and omentum, rather than spreading systemically. Pathogens introduced via the peritoneal route are rapidly sequestered by resident immune cells, including peritoneal macrophages, thereby limiting their dissemination to internal organs.^[^ ^43^, ^44^ ^]^ At 4 dpi, the virus replicated profusely in the brain tissue, particularly in the hippocampus (Figure 2g,h). Therefore, PRV Fa‐Luc can be used to visualize the replication and distribution of PRV in vivo and for further studies related to the pathogenic mechanism of PRV CNS infection.

### 2.4 CNS Infection Model of Recombinant Virus PRV Fa‐Luc

**Figure 3:**
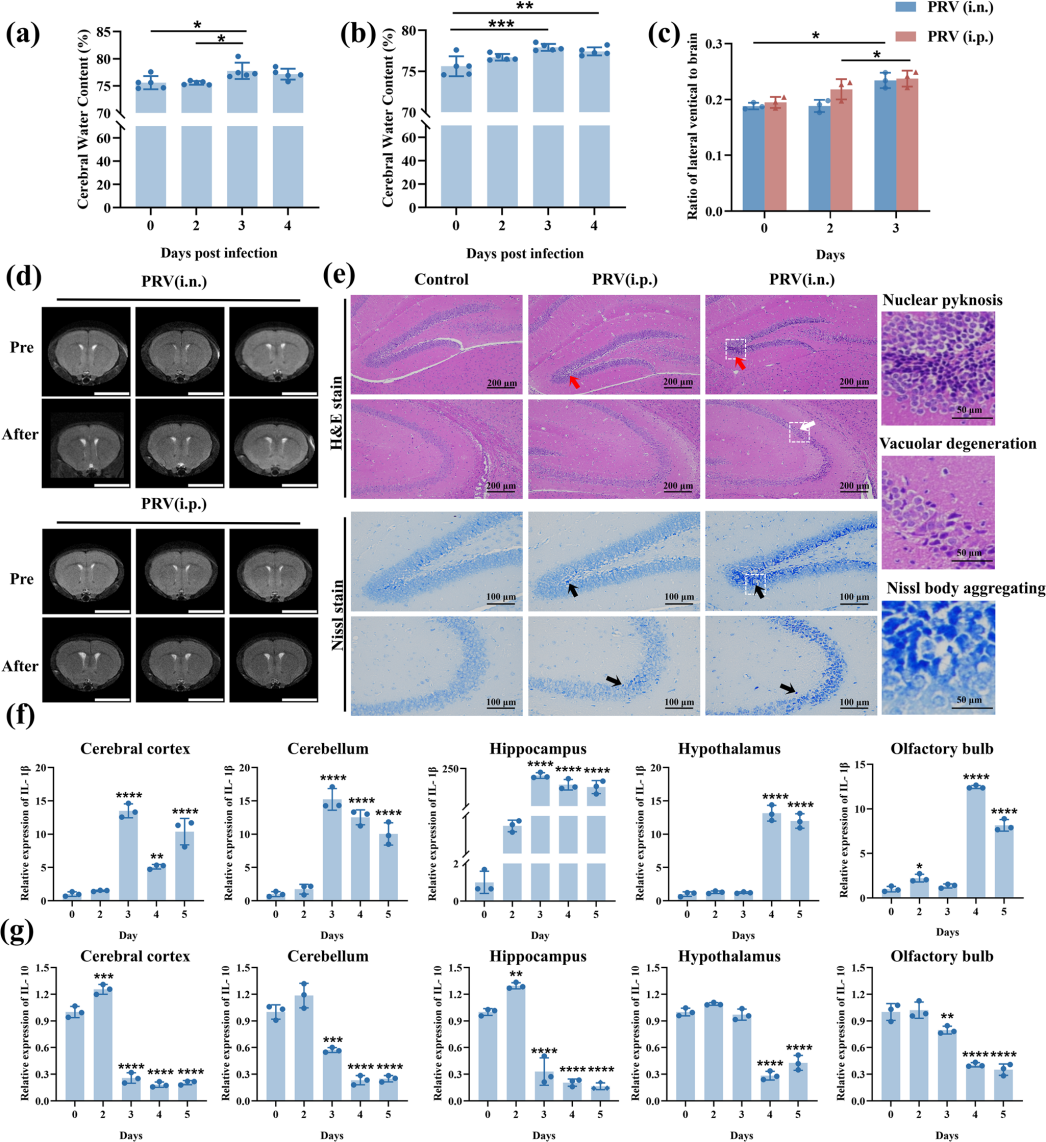
CNS infection model of recombinant virus. a,b) Cerebral water content of mice at days 0, 2, 3, 4 post intraperitoneal (a) and intranasal (b) infection of PRV (n = 5). The data for day 0 are shared between Figure 3a,b. c) Coefficient of lateral ventricles in mice (n = 3). d) Image of MRI in mice (n = 3). Scale bar: 5mm. e) Histological changes in the hippocampus area with H&E staining and Nissl staining (n = 3). Red arrows: nuclear pyknosis; White arrows: vacuolar degeneration of cells; Black arrows: Nissl body aggregating. The rightmost panels show magnified views of the areas marked by white dashed circular. f) Relative expression of IL‐1β in the brain of intranasal PRV infected mice (n = 9). g) Relative expression of IL‐10 in the brain of intranasal PRV infected mice (n = 9). Brain tissue from three mice was combined into a single independent sample in Figure 3f,g due to the small mass. Data shown as mean ± SD, One‐way ANOVA followed by Bonferroni *t*‐test in (a–c) and Dunn's post hoc test comparing all groups to the day 0 in (f) and (g). * *P*<0.05; **p < 0.01; ***p < 0.001; ****p < 0.0001.

The extent of brain tissue damage and specificity induced by intraperitoneal and intranasal infections in mice were evaluated to further validate the feasibility of establishing a CNS infection model using the recombinant PRV Fa‐Luc. The organ coefficients of the liver were significantly higher in intraperitoneally infected mice, and the organ coefficients of the lung tissue were significantly higher in intranasally infected mice, whereas none of the organ coefficients of the heart were significantly altered (Figure S1, Supporting Information). Both intraperitoneally and intranasally infected mice showed a significant increase in brain tissue water content starting on the 3 dpi after infection (**Figure** **3**a,b). MRI of the mouse brain revealed ventricular dilatation and an increase in the lateral ventricular index following infection (Figure 3c,d), thereby exacerbating PRV infection‐induced cerebral edema in mice.

Hematoxylin and eosin (H&E) staining of brain tissue was performed to examine morphological changes in neurons. In the hippocampal region, the control group exhibited normal brain tissue, characterized by round and conical neurons with distinct neuronal cell structures. In the infection group, the neuronal arrangement was sparse, with widened pericellular gaps and vacuolated degeneration. Additionally, in the CA2/3 and dentate gyrus regions, numerous cells exhibited condensed cytoplasm and nuclear pyknosis. Nissl staining was performed to measure the effects of PRV infection on neuronal viability. The nerve cells in the control group exhibited a clear structure, characterized by uniformly distributed blue granular nidus vesicles in the cytoplasm. In the intranasal infection group, the nidus vesicles were aggregated within the neurons, accompanied by intensified staining, suggesting abnormalities in neuronal structure and function. Only a few cells in the intraperitoneal injection group showed nuclear consolidation and aggregation of nidus bodies (Figure 3e).

In all groups, the olfactory bulbs exhibited a well‐preserved morphology, with the layers of the olfactory bulb maintaining a clear and intact laminar structure. Compared to the control group, the thickness of the internal plexiform layer, located between the mitral and granule cell layers, was reduced in the intranasal infection group. Under high magnification, the arrangement of internal granule cells appeared denser, and an increased quantity was observed. However, quantitative data did not show any significant differences (Figure S2a,b, Supporting Information). Changes such as perivascular lymphocyte cuff infiltration, tissue necrosis, and nucleolysis of neuronal cells were also observed in the striatum and brainstem (Figure S2a, Supporting Information), along with a significant decrease in the absolute number of nuclei and area occupied by the nuclei (Figure S2c,d, Supporting Information). These results suggest that the cell density in the striatal region was significantly reduced.

The expression levels of pro‐inflammatory factors (interleukin–1beta (IL‐1β), IL‐6, and tumor necrosis factor‐alpha (TNF‐α), anti‐inflammatory factors (IL‐10), and chemokines (monocyte chemoattractant protein‐1 (MCP‐1)) were evaluated in the brain tissues of mice from the intranasal infection group at different time points. IL‐1β content increased significantly in all regions of mouse brain tissue after infection, with peaks on 3 and 4 dpi, which were particularly evident in the hippocampus. Compared to the pre‐infection period, the olfactory bulb tissue showed a significant difference in the IL‐1β content on 2 dpi, which was earlier than in the other regions (Figure 3f). The IL‐6 and TNF‐α content similarly significantly increased. The changes of pro‐inflammatory cytokines were most significant in the hippocampus tissue (Figure S3a,b, Supporting Information). IL‐10 content increased significantly, followed by a decrease in all brain tissue regions, and its alteration was particularly significant in the cerebral cortex and hippocampus, followed by the olfactory bulb (Figure 3g). MCP‐1 was significantly altered in the olfactory bulb from the 2 dpi. In contrast, its level in the hippocampus was significantly elevated from the 3 dpi (Figure S3c, Supporting Information). These findings reveal that recombinant viruses can effectively model human PRV CNS infections, as they induce widespread inflammation in mouse brain tissue through intranasal infection.

### 2.5 Gene Expression Analysis of Brain Tissue in CNS Infection

**Figure 4:**
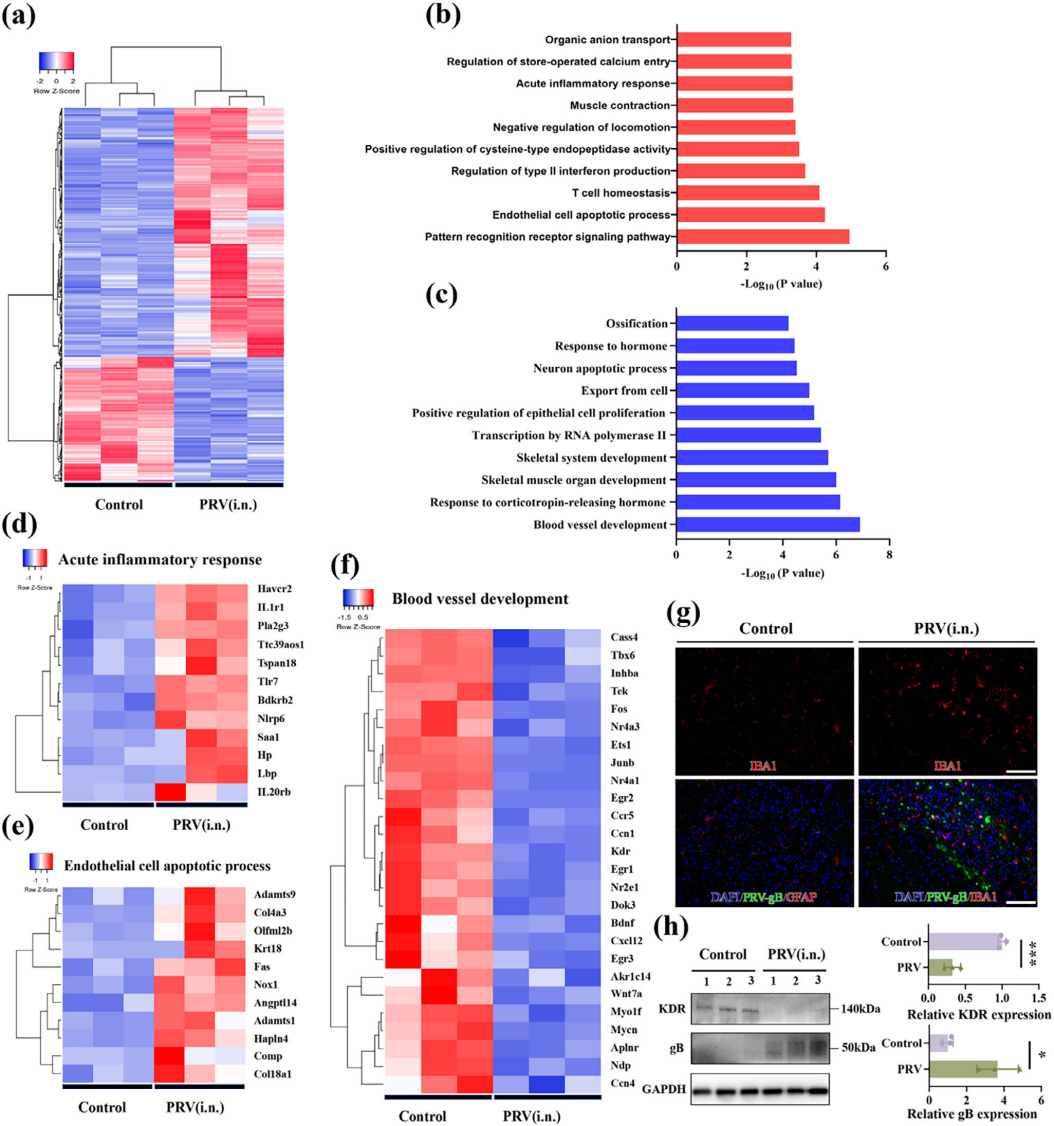
Gene expression analysis of brain tissue in CNS infection. a) Cluster heat map of DEGs (n = 3). b) Up‐regulated biological processes of DEGs in GO enrichment analysis (n = 3). c) Down‐regulated biological processes of DEGs in GO enrichment analysis (n = 3). d) Analysis of genes related to acute inflammatory response (n = 3). e) Analysis of genes related to the endothelial cell apoptotic process (n = 3). F) Analysis of genes related to blood vessel development (n = 3). g) The expression of gB and IBA1 in the brains of PRV‐infected mice and control mice was detected using immunohistochemistry. (Blue, DAPI‐indicating nuclei; Green, PRV gB; Red, IBA1). Scale bar, 100 µm. h) Detection of KDR and PRV gB protein expression in the brains of PRV‐infected and control mice by Western blotting. Data shown as mean ± SD. Quantitative analysis of protein bands using ImageJ. The significant difference between the groups was analyzed by *t* test. ***P < 0.001; *P < 0.05.

Systematic transcriptome sequencing was performed to elucidate the effect of PRV CNS infection on gene expression in the brain tissue. The gene expression of the control and PRV intranasal infection groups was classified into two major groups, and the samples within the groups exhibited the same gene expression pattern (**Figure** **4**a). Gene Ontology (GO) annotation primarily comprises three parts: biological processes (BP), cellular components, and molecular functions. As BP encompasses the core of biological activities, which are key events occurring in cells, differentially expressed genes related to BP were focused on. In the control and PRV groups, the top 10 upregulated terms of differentially expressed mRNA in GO annotation included the pattern recognition receptor signaling pathway, endothelial cell apoptotic process, T‐cell homeostasis, regulation of type II interferon production, positive regulation of cysteine‐type endopeptidase activity, negative regulation of locomotion, muscle contraction, acute inflammatory response, regulation of store‐operated calcium entry, and organic anion transport (Figure 4b). Between the control and PRV infection groups, the top 10 downregulated terms of differentially expressed mRNA in GO annotation included blood vessel development, response to corticotropin‐releasing hormone, skeletal muscle organ development, skeletal system development, transcription by RNA polymerase II, positive regulation of epithelial cell proliferation, export from cells, neuronal apoptotic process, response to hormones, and ossification (Figure 4c).

The inflammatory response plays a crucial role in innate defense mechanisms of organisms against infectious diseases.^[^ ^45^, ^46^ ^]^ Cluster analysis of genes related to the acute inflammatory response helps to identify key gene clusters that are regulated in the CNS, revealing the dynamics of the inflammatory response and its role during infection. According to the mRNA sequencing results of genes associated with the acute inflammatory response in the control and PRV intranasal infection groups, *Havcr2, IL1r1, Pla2g3, Ttc39aos1, Tspan18, Tlr7, Bdkrb2, Nlrp6, Saal, and Lbp* expression in the mouse brain was increased in the PRV intranasal infection group (Figure 4d). Endothelial cells are an essential component of the vascular wall. They are essential for vascular structure and normal physiology. The mRNA expression of endothelial cell apoptosis‐related genes was increased in mouse brain tissues after PRV CNS infection (Figure 4e). Compared with the control group, the mRNA expression of *Cass4, Tbx6, Inhba, Tek, Fos, Nr4a3, Ets1, Junb, Nr4a1, Egr2, Bdnf, Cxcl12, Ccn4, Kdr*, and other genes related to blood vessel development decreased (Figure 4f).

To validate findings from the transcriptomic analysis, we assessed the expression of viral and glial markers in the brains of PRV‐infected mice (intranasal inoculation) and controls using immunohistochemistry. Robust detection of PRV glycoprotein B (gB) in infected brains confirmed viral neuroinvasion via the olfactory route. Concurrently, significant upregulation of IBA1 (microglial marker) was observed (Figure 4g), indicating pronounced neuroinflammation triggered by PRV infection. Transcriptomic profiling revealed downregulation of vascular development genes, with KDR (a key mediator of angiogenesis) being notably suppressed. Subsequent analysis of brain homogenates by Western blot demonstrated detectable PRV gB protein in infected mice, concomitant with a significant reduction in KDR protein expression (Figure 4h). This downregulation of KDR provides mechanistic evidence that PRV infection impairs blood vessel development in the brain.

### 2.6 Cerebral Microcirculation Multimodal Imaging of CNS Infection Models

**Figure 5:**
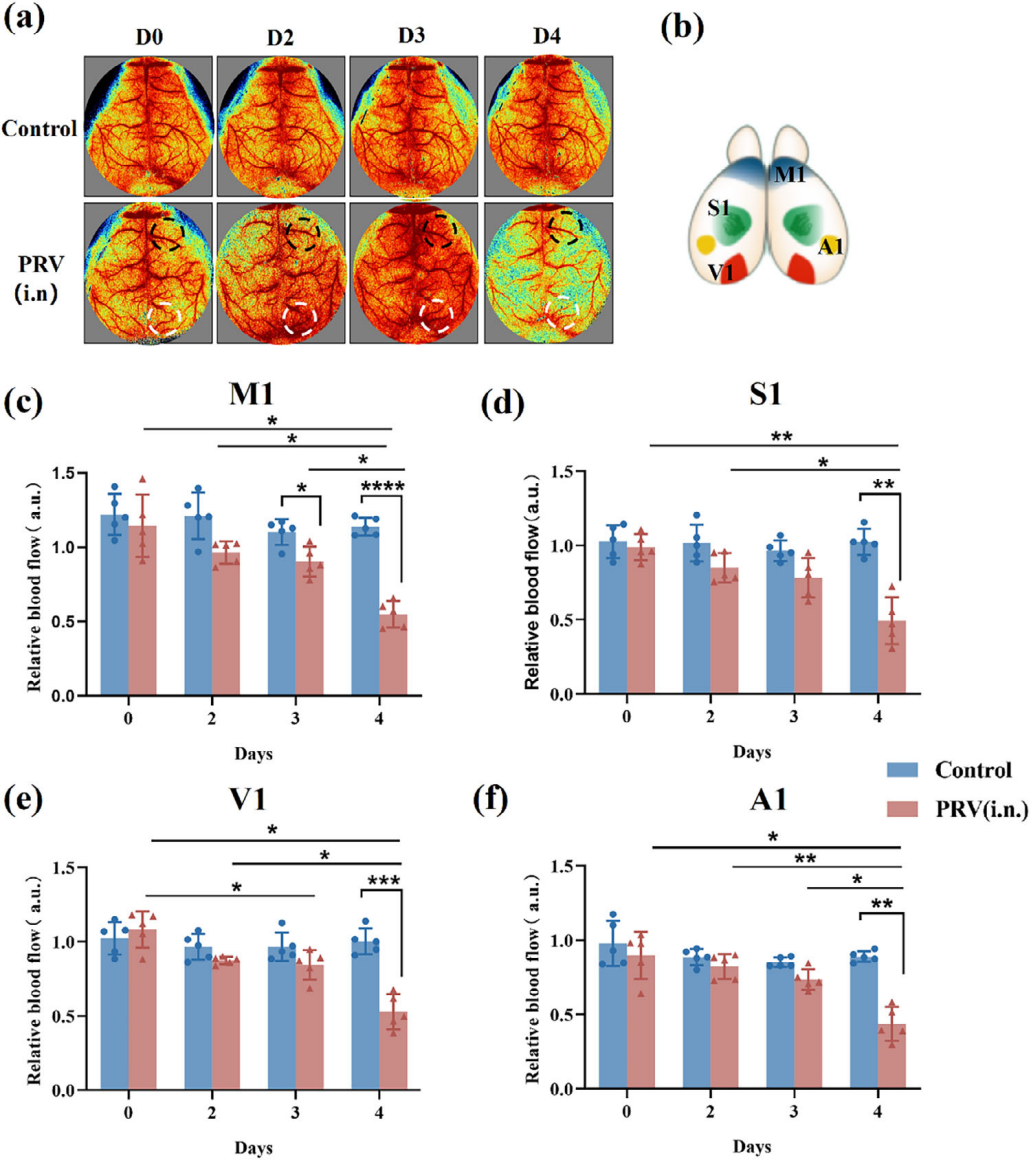
Hemodynamic monitoring of the cerebral cortex. a) LSCI images of the cerebral cortex of mice (n = 5), Black dashed circle: M1, White dashed circle: V1.(b) Schematic representation of mouse cerebral cortex partitions. c) Relative blood flow in the primary motor cortex (n = 5). d) Relative blood flow in the primary somatosensory cortex (n = 5). e) Relative blood flow in primary visual cortex (n = 5). f) Relative blood flow in primary auditory cortex (n = 5). Data shown as mean ± SD, Two‐way ANOVA in (c), (d), (e), and (f) followed by Tukey test and Šidák post hoc test comparing treatments. * *P*<0.05; **p < 0.01; ***p < 0.001; ***p < 0.0001.

The transcriptional changes observed during vascular development and endothelial apoptosis strongly suggest significant alterations in cerebrovascular function and structure. A multimodal imaging approach that integrated LSCI, OCTA, and two‐photon imaging was employed to simultaneously monitor changes in cerebral blood flow and vascular structure and to directly visualize and quantify these effects. This powerful combination enhances imaging sensitivity, allowing for a comprehensive understanding of the dynamic effects of the virus on cerebrovascular mechanisms at various stages of infection. To investigate cerebral vascular abnormalities during CNS infection, cerebral blood flow in mice was monitored using the LSCI system at 0, 2, 3, and 4 dpi (**Figure** **5**a), and relative blood flow (RBF) was measured in specific regions of interest, including the primary motor cortex (M1), primary somatosensory cortex (S1), primary visual cortex (V1), and primary auditory cortex (A1) (Figure 5b–f). As highlighted by the dashed circular annotations in Figure 5a, signs of cortical hyperemia appeared as early as 2 dpi, particularly in the M1 and V1 regions, and subsided by 4 dpi. In addition, changes in RBF became apparent at 3–4 dpi, with a pronounced reduction in all cortical regions by 4 dpi, especially in the annotated M1 and V1 areas (Figure 5c–f). The sharp decline in blood flow throughout the entire dorsal cortex may be attributed to the direct effect of the virus on endothelial cells, resulting in vascular dysfunction and inflammation. Additionally, changes in the metabolic demands and neuronal dysfunction in these areas may contribute to reduced blood flow.

**Figure 6:**
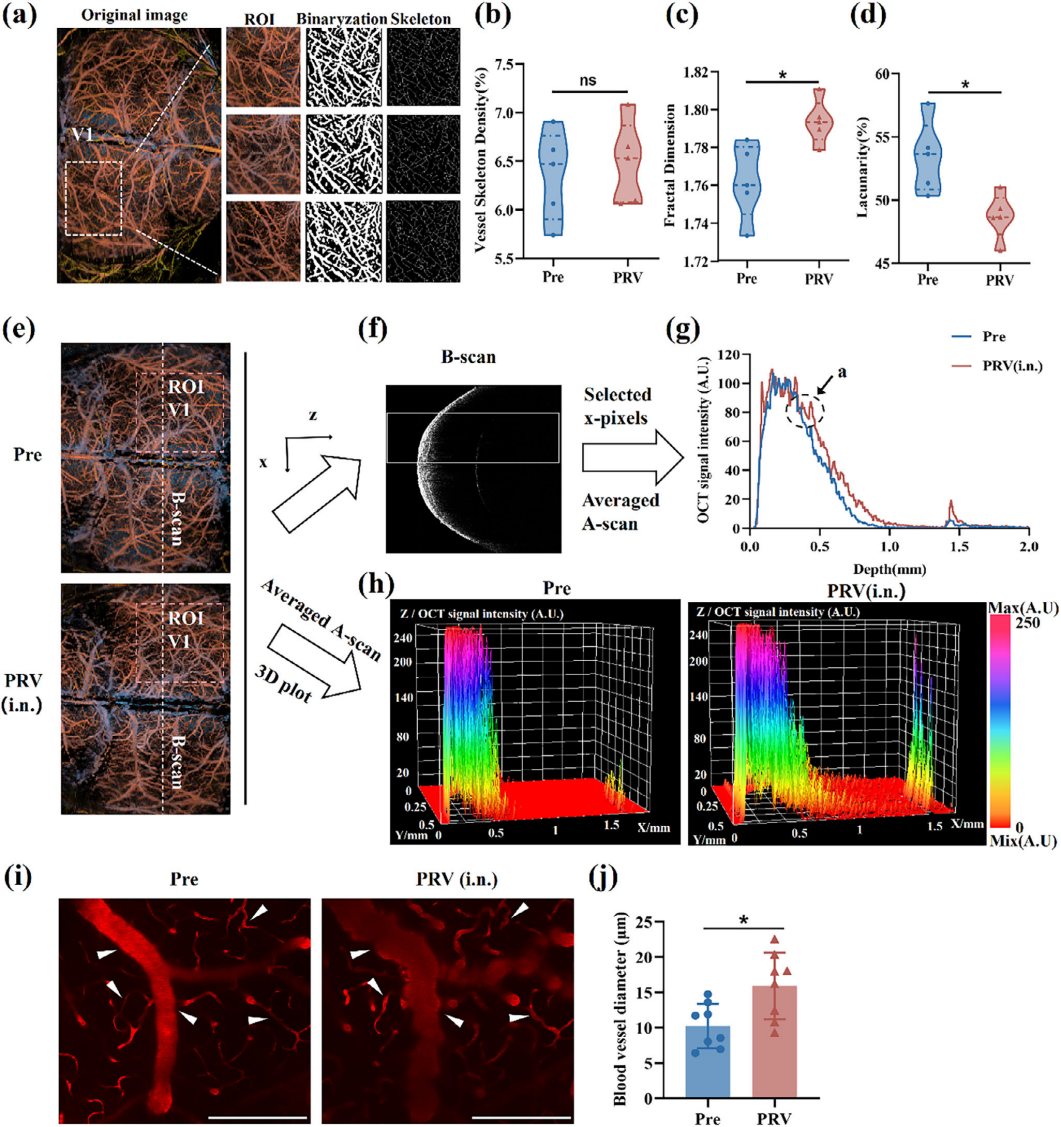
Vascular network analysis of primary visual cortex. a) Representative OCTA images from three individual mice showing the vasculature of V1. b) Vascular skeleton density of V1 (n = 3). c) Fractal dimension of vascular network of V1 (n = 3). d) Lacunar degree of vascular network of V1 (n = 3). e) Map of OCT signal extraction area (n = 3). f) B‐scan at the position of the white line in A, the X axis and Y axis represent the imaging depth and the OCT signal intensity (n = 3). g) 2D OCT signal extracted from e. h) 3D OCT signal distribution map of V1 (n = 3); 3D OCT signal extracted from the ROI region of e, X axis and Z axis indicates imaging depth and OCT signal. i) Images of blood vessels in V1, white arrows: vasodilation, scale bar: 200 µm (n = 3). j) Quantification of vessel diameter within the 0–150 µm depth range in the V1 region (n = 3; 2 – 3 vessels per mouse with baseline diameter of 5–15 µm), (n = 3). Data shown as mean ± SD, Paired two‐tailed Student's t‐test in (b), (c), (d), and (j). ns, no significance; * *P*<0.05.

Considering the ocular inflammation induced by human PRV infection^[^ ^47^, ^48^ ^]^ and the pronounced blood flow alterations observed in the V1 region after LSCI, the effects of PRV infection on vascular architecture within the V1 area were investigated. The skeleton of the vascular network in V1 of brains collected from mice before and after infection was extracted, and various vascular parameters were quantified. After in vivo OCTA scanning of the mouse brain, color‐coded maps of the cerebral vasculature OCTA with depths ranging, as well as 3D hierarchical images, were initially extracted based on the 800 B‐can images obtained. The vascular network was further reconstructed using the Frangi 2D filter based on the Hessian matrix, and suitable thresholds were selected to construct a binary map of the vascular region. The vascular skeleton map and the vascular edge information map were then constructed using the binary map (Figure S4, Supporting Information). Compared to the pre‐infection period, the density of the vascular skeleton in V1 of CNS‐infected mice did not show significant changes; however, the fractal dimension of the vascular network was significantly elevated, and the luminal dimension was significantly decreased (**Figure** **6**a–d).

The B‐scan images of V1 were extracted, and the OCT signal intensity was analyzed at different depths to further assess the depth of the vascular lesions. First, a single B‐scan image from the same location in V1 was randomly selected before and after infection (Figure 6e), and the OCT signals from a depth of 0 to 2 mm were compared (Figure 6f). Tissue signal intensity significantly increased starting from point a post‐infection (Figure 6g), indicating structural changes in the brain tissue. Additionally, OCT signals from all 200 B‐scans of V1, covering depths of 0–2 mm, were extracted to generate a 3D OCTA signal intensity distribution (Figure 6h). The analysis revealed a notable signal enhancement starting at ≈500 nm, which was consistent with the findings of the single B‐scan analysis.

Next, the change in vessel diameter within V1 at depths ranging from 0 to 150 µm after skull removal was evaluated. Two‐photon microscopy revealed significant dilation and swelling in this region following CNS infection, characterized by irregular surface protrusions and increased structural complexity compared to the healthy state (Figure 6i). Additionally, the vessel diameter increased significantly (Figure 6j), indicating that CNS infection may induce abnormal vascular dilatation.

Thus, PRV CNS infection induces cerebrovascular dysfunction through the activation of pattern recognition receptor signaling pathways (e.g., *TLR7*) and acute inflammatory response genes (e.g., *Havcr2* and *IL1r1*), the upregulation of endothelial apoptosis‐related pathways (e.g., caspase activity regulation), and the suppression of vascular development genes (e.g., *Kdr, Cxcl12*, and *Tek*). These molecular alterations drive neuroinflammatory storms, vascular endothelial damage, and impaired angiogenesis, culminating in cerebral circulatory disturbances characterized by early‐stage hyperemia and late‐stage hypoperfusion, with compensatory vascular network remodeling.

## 3 Discussion

The emergence of zoonotic neurotropic viruses presents a significant public health challenge, with neuroinfectious diseases caused by these viruses becoming increasingly concerning.^[^ ^49^, ^50^ ^]^ However, understanding the mechanisms of infection and host responses remains a major challenge. To address these gaps, the EP0‐sgRNA‐hSPCas9 gene‐editing system was utilized to insert green fluorescent protein and firefly luciferase expression cassettes into the PRV genome, resulting in the creation of recombinant virus PRV Fa‐Luc. This approach enabled the in vivo tracking of the virus, highlighting its differential tissue tropism and replication dynamics. Upon intraperitoneal infection, the virus primarily accumulates in the liver, whereas intranasal inoculation leads to early enrichment in the lungs, followed by replication in the brain tissue at 3–4 dpi, triggering inflammation and neuronal damage. Notably, the hippocampus showed more severe viral accumulation and damage than the other brain regions, whereas the cerebral cortex also exhibited signs of viral dissemination, demonstrating the potent neuroinvasive potential of the virus. This recombinant virus enables the precise tracking of viral distribution, offering a tool for studying viral behavior and pathogenesis across various infection routes. Notably, this gene‐editing strategy can be adapted to generate recombinant versions of various neurotropic viruses, facilitating the study of virus‐host interactions and disease mechanisms in diverse biological systems.

The cerebral microcirculation plays a crucial role in brain function, and its disruption severely impairs neural activity.^[^ ^51^, ^52^, ^53^ ^]^ The CNS relies on the integrity of its vascular network during development, tissue homeostasis, and disease recovery.^[^ ^54^, ^55^ ^]^ Neurotropic viral infections, such as PRV, induce substantial alterations in the cerebrovascular structure and function. In this study, an early congestive response was observed in cerebral cortical blood flow, followed by hemodynamic adjustments. Initial congestion may be attributed to infection‐induced inflammation in which neuronal damage and immune cell activation lead to the release of inflammatory mediators, resulting in vasodilation and increased vascular permeability.^[^ ^56^, ^57^ ^]^ The subsequent decline in blood flow likely reflects the direct effect of the virus on the vascular system or the further development of inflammatory responses.^[^ ^58^, ^59^ ^]^ These findings highlight the complex regulatory processes involved in the CNS responses to viral infections.

Additionally, PRV infection leads to vascular dilation and deformation in V1, resulting in an increased complexity of the vascular network. This was evidenced by OCTA analysis, which revealed a significantly increased fractal dimension and decreased lacunarity despite no significant change in vascular skeleton density. This apparent inconsistency is likely due to the limitations of OCTA in detecting changes in vessel diameter. Furthermore, two‐photon vascular imaging confirmed a significant post‐infection increase in vessel diameter, suggesting that vascular remodeling primarily involves vessel dilation rather than angiogenesis. These findings suggest that single‐modality imaging may not fully capture all relevant aspects of cerebrovascular alterations, emphasizing the need for multiple imaging modalities to comprehensively characterize virus‐induced vascular remodeling.

This multi‐modality imaging approach, which combines various imaging techniques, provides high‐resolution spatiotemporal insights into cerebral blood flow, vascular permeability, and neurovascular interactions following PRV infection. These findings revealed significant alterations in microvascular perfusion and endothelial integrity, which correlated with the spread of the virus and the development of neuroinflammatory responses. This comprehensive approach provides novel insights into cerebrovascular pathology associated with neurotropic viral infections and highlights the potential of multi‐modality imaging to investigate virus‐induced neurovascular dysfunction.

Transcriptomic sequencing, combined with immunohistochemical and molecular validation, further corroborated these findings, suggesting that PRV infection impairs normal vascular development. The activation of immune cells and the release of inflammatory factors during CNS infection lead to endothelial dysfunction and apoptosis, inhibiting vascular development. This disruption in vascular formation exacerbates blood supply deficits and metabolic imbalances, ultimately hindering neuronal regeneration and repair, potentially leading to persistent neurological dysfunction or neurodegeneration. By integrating multiple imaging modalities, the hemodynamic changes and compensatory responses in the vascular network can be evaluated more effectively, providing a deeper understanding of the interactions between vascular integrity, inflammation, and neuronal health. Although this study provides valuable insights into how neurotropic viruses affect cerebral microcirculation, further methodological advancements are required to assess the long‐term effects of these infections on cerebrovascular function and brain health. Additionally, rodents are widely used in viral pathogenesis research; however, their microvascular architecture differs from that of humans, which may result in discrepancies in findings. Future studies should focus on developing more accurate animal models of viral infections that replicate human neurovascular complications and disease progression.

In conclusion, an intranasal mouse model of double‐labeled recombinant PRV Fa‐Luc effectively simulated a human CNS infection. Combined with multi‐modal angiography, it enables visualization of cerebral vascular abnormalities during neurotropic viral infections, providing a powerful tool for investigating the pathological mechanisms of human neurotropic viral infections and advancing vaccine development. With further optimization, this model, when integrated with multi‐modal imaging technology, can become a crucial platform for studying cerebrovascular and neurotrophic diseases.

### 3.1 Advantages and Limitations

Although previous studies have shown that neurotropic viruses, including varicella‐zoster virus,^[^ ^60^, ^61^ ^]^ zika‐virus,^[^ ^62^, ^63^, ^64^ ^]^ and COVID‐19^[^ ^65^ ^]^ can compromise cerebrovascular integrity through mechanisms such as endothelial damage, vascular malformations, and microhemorrhages, these findings predominantly rely on histological analyses or single imaging modalities with limited spatial and temporal resolution. Therefore, a comprehensive understanding of the dynamic vascular changes that occur during infection is limited, particularly during the early stages of infection.

This study addressed these gaps by generating a recombinant PRV Fa‐Luc using a CRISPR‐based genome editing system and by establishing an intranasal infection model that mimics the key features of CNS infection. A multiple‐modality imaging strategy was employed that combined macroscale MRI and bioluminescence with microscale LSCI, OCTA, and two‐photon microscopy to achieve real‐time, multiscale visualization of cerebrovascular responses during infection. Compared with traditional single‐modality approaches, this method offers superior resolution, imaging depth, and temporal dynamics, enabling a more comprehensive characterization of virus‐induced cerebrovascular changes.

However, this study has several limitations. The mouse brain microvasculature differs from that of humans, which may limit its translational relevance. Additionally, although this imaging strategy bridges multiple scales, the visualization of deeper brain regions remains limited, and further standardization is required to fully integrate multimodal datasets. Future studies should incorporate larger animal models and advanced imaging systems to better replicate and interpret human neurovascular pathologies.

## 4 Materials and Methods

### Virus and Cells

The PRV wild‐type strain Fa was grown in PK‐15 cells purchased from ATCC (ATCC CCL‐33). The viral titer was determined by plaque assay. 293β5 cells and MDCK cells were gifts from Prof. Quan Yuan of Xiamen University and from Prof. Honglin Chen of Hongkong Kong University. Panc‐1 cell line was purchased from ATCC (ATCC CCL‐1469). GBM cell line was isolated from human glioblastoma tissue.^[^ ^66^ ^]^ All cells were grown in Dulbecco's modified Eagle's medium (DMEM) (Invitrogen, USA) supplemented with 10% heat‐inactivated fetal bovine serum (FBS) (Gibco, USA), 100 µg/mL streptomycin, and 100 IU mL^−1^ penicillin at 37 °C in a humidified 5% CO_2_ atmosphere.

### Animals

Specific pathogen‑free (SPF) BALB/c female mice (age, 6–8 weeks; weight, 18–20 g) were purchased from Shanghai SLAC Laboratory Animal Co., Ltd. The room temperature was maintained at 25±2  °C with 60%–70% relative humidity. The circadian rhythm was set to 12 h, and food and water were supplied freely. Mice were humanely euthanized when body weight fell below 75% of baseline or upon reaching predefined experimental endpoints. All protocol in this study was approved by the Committee on the Ethics of Animal Care and Use of Xiamen University. The study was conducted following the Guide for the Care and Use of Animals in Research of the People's Republic of China (XMULAC20230320).

### Construction of EP0‐sgRNA‐hSPCas9

The EP0‐sgRNA‐hSPCas9 plasmid was constructed by first performing double digestion of the hspCas9 plasmid using Xba I and Age I restriction enzymes. Two U6p‐EP0‐sgRNA expression frames were then obtained through PCR amplification of the hspCas9 plasmid, using the primers U6P‐F, U6P‐R1 for the first sgRNA scaffold and U6p‐R2 for the second. The three fragments, the digested hspCas9 plasmid, and the two sgRNA expression frames—were then recombined by Gibson assembly, which facilitated the seamless joining of the fragments. The resulting recombinant plasmid was validated through sequencing to confirm the correct insertion and orientation of the sgRNA sequences, making it ready for use in genome editing experiments targeting the EP0 gene.

### Construction of Donors

The target fragment for homologous recombination was obtained by PCR using the PTT5‐luciferase plasmid as a template. To create the donor plasmid, the original plasmid was double‐digested using the restriction enzymes Bgl II and Xho I, generating a 5000 bp fragment that was recovered for further use. The target fragment and the digested plasmid were then combined using Gibson assembly, a process facilitated by incubating the mixture in a water bath at 55 °C for 1 h. This step created the Donor plasmid, which was prepared for use in subsequent homologous recombination experiments.

### Homologous Recombination mediated by CRISPR‐Cas9

293β5 cells were seeded into 24‐well plates. Upon reaching 70%–90% confluency, the cells were co‐transfected with the plasmids EP0‐sgRNA‐hSPCas9 and a donor plasmid using Lipofectamine 3000 (Thermo Fisher Scientific, L3000015) according to the manufacturer's protocol. Twenty‐four hours post‐transfection, the cells were infected with PRV‐Fa strain at a multiplicity of infection (MOI) of 0.1. The supernatant was harvested 48 h post‐infection. Subsequently, 50 µL of the supernatant was used to infect Panc‐1 cells. Twenty‐four hours later, mNeonGreen (mNG)‐positive Panc‐1 cells were isolated via single‐cell fluorescence‐activated cell sorting (FACS) and co‐cultured with pre‐seeded GBM cells in 96‐well plates. mNG‐positive cells in the 96‐well plates were monitored using a high‐content imaging system. Supernatants from wells exhibiting positive signals were collected for subsequent viral validation, purification, and propagation. Sorted cells were co‐cultured with pre‐seeded glioblastoma (GBM) cells in 96‐well plates to facilitate viral transfer. Virus particles released from infected GBM cells were collected, expanded, and utilized for subsequent experiments.

### In vitro and In vivo Assessment of Firefly Luciferase Enzyme Activity

2 × 10 ^4^ GBM cells were inoculated in 96‐well plates infected with PRV Fa‐Luc at different MOIs. According to the One‐Lite Luciferase Assay System kit (Nanjing Vazyme Biotech Co. Ltd., Nanjing, China), the luciferase activity was detected in each well at different time points. At 0, 2, 3, and 4 days after PRV infection, mice were anesthetized with isoflurane. Liver, lung, heart, and brain tissues were removed and mechanically homogenized in 0.9% normal saline at 200 mg ml^−1^ (cerebral cortex, hippocampus, cerebellum, hypothalamus, and olfactory bulb tissues were combined as an independent sample to prepare tissue homogenates due to their small mass). The tissue homogenate was aliquoted into a 96‐well plate, and Luciferase enzyme activity was measured in each well at various time points using the One‐Lite Luciferase Assay System kit (Nanjing Vazyme Biotech Co. Ltd., Nanjing, China).

### Western Blot

The cell culture medium was removed, and the cells were washed three times with ice‐cold phosphate‐buffered saline (PBS). RIPA lysis buffer (Beyotime, P0013B) supplemented with 1 mM PMSF(Beyotime, ST507)was then added to lyse the cells. After complete lysis, sodium dodecyl sulfate (SDS) sample loading buffer was added, and the samples were denatured by incubation at 95 °C for 15 min. The membrane was blocked at room temperature for 30 min with blocking buffer containing 5% bovine serum albumin (BSA). Primary antibody incubation was performed overnight at 4 °C. After washing the membrane three times with washing buffer, secondary antibody incubation was conducted at room temperature for 1 h. Protein signals were visualized using enhanced chemiluminescence (ECL) reagent and imaged by Fusion FX7 Edge.

Brain tissues were homogenized in 1 mL ice‐cold phosphate‐buffered saline (PBS) using a mechanical homogenizer. A 50‐µL aliquot of the homogenate was mixed with 1 mL of RIPA lysis buffer (containing 1 mM PMSF protease inhibitor) and vortexed thoroughly. The mixture was sonicated on ice (3 × 5‐sec pulses at 30% amplitude) to ensure complete cell lysis. Lysates were centrifuged at 12000 rpm for 15 min at 4 °C. The supernatant was collected into fresh microcentrifuge tubes and subjected to Western blot analysis. GAPDH Monoclonal antibody (Proteintech, 60004‐1‐Ig). Anti‐KDR antibody (Cell Signaling Technology, 9698S).

### Mouse Survival Study

To evaluate the virulence of the recombinant virus PRV Fa‐Luc, a survival study was conducted using 30 healthy 6‐week‐old female BALB/c mice. The mice were randomly assigned to five groups, each consisting of 5 mice. The experimental groups were inoculated with varying doses of PRV Fa‐Luc via either intraperitoneal (i.p.) or intranasal (i.n.) routes. The doses administered were 13 pfu, 25 pfu, 50 pfu, 100 pfu, 200 pfu, and 400 pfu, with 100 µL administered for the i.p. injection and 50 µL for the i.n. inoculation. Following infection, the mice were monitored daily for clinical signs of illness, including any changes in behavior, posture, or overall health. Mortality was recorded, and the survival rate was determined over a 7‐day period. Additionally, the body weight of each mouse was measured daily to assess the impact of the virus on general health and to track any significant weight loss that could indicate the severity of infection.

### Grouping and modeling animals

The mice were randomly assigned to one of three experimental groups, each consisting of five mice, to ensure unbiased results:^[^ ^1^ ^]^ the control group,^[^ ^2^ ^]^ the intraperitoneal injection group (i.p.), and^[^ ^3^ ^]^ the intranasal injection group (i.n.). This randomization helped to minimize any potential confounding variables that could arise from systematic biases in group selection. Each group was subjected to its respective treatment to model the different routes of infection and examine the effects of the infection over time. At specified time points—0‐, 2‐, 3‐, and 4‐dpi—mice from each group were euthanized, and their brain tissues were carefully harvested for further analysis. The tissues were processed for tissue sectioning to enable detailed histological examination and for RNA and protein extraction to facilitate molecular analyses.

### Bioluminescence Imaging

Bioluminescence imaging was performed at 0, 2, 3, and 4 days after PRV intraperitoneal or intranasal infection to trace the localization and progression of the virus in vivo. Mice were anesthetized with isoflurane, first induced at 2‐2.5% and then maintained at 1%–1.5% in oxygen at a flow rate of 0.8 L min^−1^. To facilitate viral detection, 200 µL of D‐luciferin sodium salt (150 mg kg^−1^ body weight, 15 mg mL^−1^) was intraperitoneally injected. D‐luciferin serves as the substrate for firefly luciferase expressed by the recombinant PRV, enabling real‐time visualization of viral distribution through light emission. 5 min after injection, the mice were placed in the Caliper IVIS Lumina II imaging chamber for bioluminescence detection. The imaging system collected signals using a full reception filter, with an exposure time of 30 s to capture optimal signal intensity.

### Organ Coefficient Measurement

Mice were anesthetized after measuring body weight (BW). Liver, lung, and heart tissues were removed and immediately weighed (organ weight, OW). The organ coefficient was calculated as (organ weight/body weight) × 100%.^[^ ^67^ ^]^

### Brain Edema

Mice were anesthetized after imaging. Brain tissues were removed and immediately weighed (wet weight, WW), and then dried for 72 h at 80 °C to obtain dry weight (DW). The percentage of brain water content (BWC) was calculated as [(wet weight − dry weight)/wet weight] × 100%.

### Magnetic Resonance Imaging

Magnetic resonance imaging (MRI) was performed using a Bruker 9.4 T Micro MRI system to assess brain changes at 0, 2, 3, and 4 days following PRV intraperitoneal or intranasal infection. Mice were first anesthetized with an appropriate anesthetic protocol to ensure deep sedation and minimize movement during imaging. The animals were then positioned with their heads secured inside the radiofrequency coil of the MRI scanner to achieve optimal image quality. The MRI parameters were carefully optimized for this study. The field of view (FOV) was set to 2.5 cm × 2.5 cm, ensuring adequate coverage of the brain regions of interest. The repetition time (TR) and echo time (TE) were set to 2262 ms and 33 ms, respectively, to achieve a good balance between image resolution and signal‐to‐noise ratio. A total of 25 layers (slices) were acquired, with each slice having a thickness of 0.5 mm, allowing for fine resolution of brain structures. The scan was conducted without any slice spacing to provide contiguous, high‐quality cross‐sectional images of the brain. The resulting MRI images were analyzed to observe structural changes in brain regions affected by the PRV infection, particularly focusing on areas such as the cortex, hippocampus, and other critical regions involved in the pathology. The high‐resolution imaging provided valuable insights into the progression of the infection and its impact on brain tissue over time.

### Histological Examination

The brain tissue samples of mice were carefully harvested and immediately fixed in 4% paraformaldehyde at 4 °C for 24 h to preserve cellular morphology. After fixation, the tissue was dehydrated through a graded series of ethanol solutions and subsequently embedded in paraffin to facilitate sectioning. Once embedded, 4 µm‐thick tissue sections were prepared using a microtome. For each brain region, three consecutive sections were obtained per sample (n = 3 mice per group). The anatomical coordinates of the analyzed regions were determined based on The Mouse Brain in Stereotaxic Coordinates, Third Edition (Paxinos and Franklin), as follows:

Olfactory bulb: Interaural 8.08 mm; Bregma 4.28 mm

Striatum: Interaural 4.78 mm; Bregma 0.98 mm

Hippocampus: Interaural 1.98 mm; Bregma –1.82 mm

Brainstem: Interaural –2.08 mm; Bregma –5.88 mm

These sections were subsequently subjected to either hematoxylin and eosin (H&E) staining for the assessment of general tissue morphology or 0.2% Toluidine Blue staining (Solarbio, China) for the visualization of Nissl bodies, enabling the identification of specific cellular components such as neurons and glial cells. After staining, all sections were examined using a light microscope to evaluate histological architecture and detect any pathological alterations.

### ELISA

After imaging, mice were anesthetized, and the brain tissues were removed. The brain tissues were mechanically homogenized in 0.9% normal saline at 200 mg/ml and centrifuged at 12000 rpm for 10 min at 4 °C. (Due to the limited tissue mass obtained from individual mice for cerebral cortex, hippocampus, cerebellum, hypothalamus, and olfactory bulb, tissues from three mice were pooled to constitute a single independent sample for tissue homogenate preparation). The concentrations of IL‐1β, IL‐6, TNF‐α, IL‐10, and MCP‐1 in brain tissue homogenates were quantified using specific enzyme‐linked immunosorbent assay (ELISA) kits for mice (Beyotime, Shanghai, China) according to the manufacturer's instructions. The final concentration of cytokines was measured using OD values. Brain tissue from three mice was combined into a single independent sample due to the small mass.

### Laser Speckle Contrast Imaging of the Cerebral Cortex

At 0, 2, 3, and 4 days after PRV intranasal infection, mice were anesthetized using an appropriate anesthetic regimen, ensuring that they remained stable and unconscious throughout the procedure. Cerebral blood flow was monitored using a laser speckle flow contrast imaging system, which provides a non‐invasive method for assessing regional blood flow in the brain. The system used a laser with a wavelength of 780 nm, allowing for high‐resolution imaging of blood flow dynamics. The imaging system was equipped with a CMOS sensor with a resolution of 1472 × 1104 pixels, enabling detailed capture of the blood flow patterns across the brain. The field of view covered ≈12 × 15 cm, and imaging was conducted at a frame rate of 20 fps to capture rapid changes in blood flow. Mice were placed securely on a small animal adapter, ensuring that the head was fixed in place to avoid any movement during the imaging process. Vessel images were then acquired, focusing on key regions of interest as defined by the mouse brain atlas. Regions including the primary motor cortex, primary somatosensory cortex, primary visual cortex, and primary auditory cortex were specifically selected for blood flow analysis. These regions were chosen due to their involvement in sensory and motor functions, which may be impacted by the PRV infection. The relative blood flow in each of these regions was quantified and compared across the different time points. After completing the imaging session, the skin was carefully sutured to close the incision, and the area was sterilized to prevent infection. All procedures were conducted in accordance with animal welfare guidelines to minimize discomfort and ensure the well‐being of the mice.

### OCTA Imaging of the Primary Visual Cortex

To achieve high‐precision, noninvasive monitoring of the orthotopic visual cortical vascular network in PRV intranasally infected mice, a self‐built swept‐source optical coherence tomography (OCT) system was utilized. The system employed a high‐speed swept‐source laser (Axsun 105, AXSUN Technologies Inc., Billerica, MA) with a central wavelength of 1060 nm, a sweep frequency of 200 kHz, and a full width at half maximum (FWHM) of 100 nm. During imaging, the field of view (FOV) was 10 mm × 10 mm. Each B‐scan consisted of 400 A‐lines, and each C‐scan comprised 400 B‐scans. To improve signal quality and motion contrast, each B‐scan location was scanned four times. This high‐speed, high‐resolution laser system allowed for detailed imaging of the vascular structures within the primary visual cortex, providing invaluable insights into the changes induced by the PRV infection over time. At 0‐, 2‐, 3‐, and 4‐dpi, mice were anesthetized with an appropriate regimen to ensure deep sedation, reducing movement during the imaging process. They were then carefully placed on a small animal adapter, ensuring their head remained in a fixed, horizontal position to maintain consistency across imaging sessions. The vessel images were subsequently acquired, focusing specifically on the primary visual cortex, a key region affected in the PRV infection model. Changes in primary visual cortex vessel morphology were quantified using the OCTAVA algorithm on the MATLAB (MathWorks Inc., Natick, Massachusetts) platform.^[^ ^55^ ^]^

### Two‐Photon Imaging of Primary Visual Cortex

4 days after PRV intranasal infection, mice were anesthetized and placed securely on a small animal adapter, keeping their heads fixed in a horizontal position to ensure stability during the imaging process. The skull was exposed, and a circular craniotomy (4 mm diameter) was performed over the primary visual cortex using a high‐speed dental drill. After the dura was carefully removed, a glass cover slip was placed on the exposed cortical surface and sealed with glue. A custom head plate was then secured to the skull with dental cement, providing a stable platform for the mouse during imaging. To visualize the cortical blood vessels, Texas Red dextran (70 kDa) was administered intravenously, allowing for clear identification of the vasculature.

Two‐photon imaging was employed to analyze the cortical vessels, utilizing an excitation wavelength of 920 nm to optimize tissue penetration and fluorescence excitation. The imaging was conducted in two modes: line scan mode to measure blood flow velocity by tracking the movement of the fluorescent dextran through the vessels, and Z‐sequence 3D scan mode for multi‐slice scanning to capture the full 3D structure of the vascular network. The scanning resolution was set to 1024 × 1024 pixels, with a scanning speed of 2.0 µs pixel^−1^ and a z‐axis step size of 5 µm, allowing for high‐resolution, dynamic visualization of the cortical vasculature. This approach provided detailed, non‐invasive monitoring of the vascular changes induced by the PRV infection.

### Transcriptome Sequencing and Analysis

Brain tissue samples were harvested after transcardial perfusion with cold PBS to minimize blood contamination. Total RNA was extracted using TRIzol reagent, and RNA integrity was confirmed by Bioanalyzer. mRNA was then enriched using poly(A) selection and reverse‐transcribed to generate cDNA libraries. High‐throughput sequencing was performed using the Illumina NovaSeq platform, yielding paired‐end 150 bp reads. Raw sequencing data underwent quality control using FastQC, and low‐quality reads and adaptors were removed using Trimmomatic. Clean reads were aligned to the reference genome using HISAT2. Gene‐level read counts were obtained with HTSeq‐count, and expression levels were normalized and calculated as fragments per kilobase per million mapped reads (FPKM). Differential expression analysis was performed using the DESeq2 R package. Genes with an adjusted p‐value (Padj) ≤ 0.05 and |log2 fold change| ≥ 1 were defined as differentially expressed genes (DEGs). These DEGs were subjected to Gene Ontology (GO) enrichment and Kyoto Encyclopedia of Genes and Genomes (KEGG) pathway analyses to identify infection‐associated biological functions. In particular, genesrelated to acute inflammatory response, endothelial cell apoptotic process, and blood vessel development were analyzed in detail and visualized using clustering heatmaps.^[^ ^68^, ^69^, ^70^, ^71^ ^]^

### Immunofluorescence STAINING

Mouse brains were fixed in 4% paraformaldehyde at 4 °C for 2 h, followed by sequential dehydration in 10%, 20%, and 30% sucrose solutions. Tissues were embedded in OCT and cryosectioned at 50 µm. Sections were dried at 55 °C for 30 min, then washed three times with PBS (5 min each), permeabilized with 0.3% Triton X‐100 for 15 min at room temperature, and blocked with 3% BSA for 1 h. Primary antibodies (IBA1, Oasis Biofarm, OB‐PRB029‐02, 1:1000; anti‐PRV gB antibody, 1:1000) were applied and incubated overnight at 4 °C. After three PBS washes, sections were incubated with fluorescent secondary antibodies (Goat anti‐Human, Thermo Fisher Scientific, A11013, 1:200; Alexa Fluor 647‐conjugated AffiniPure Goat Anti‐Rabbit IgG (H+L), Jackson, AB_2 338 072, 1:200) for 2 h at room temperature, followed by DAPI staining (R37606) for 10 min. After final PBS washes, sections were mounted and imaged using a Leica DM6B microscope. The anatomical coordinates of the analyzed regions were determined based on The Mouse Brain in Stereotaxic Coordinates, Third Edition (Paxinos and Franklin), as follows:

Brainstem: lLateral 0.48 mm; Bregma –7 mm.

### Statistical analysis

Statistical analysis was performed using GraphPad Prism 9 software (GraphPad Inc., San Diego, CA, USA), and data are presented as the mean + standard error of the mean. Statistical analysis of data was performed with the one‐way ANOVA and the Two‐way ANOVA. A paired two‐tailed Student's T‐test was used to analyze the significance between the two groups. Statistical significance was indicated as *P < 0.05; **P < 0.01; ***P < 0.001; ****P < 0.0001; ns, no significance. Experimental data were obtained from at least three independent replicates. Statistical parameters, including statistical analysis, statistical significance, and sample sizes (denoted as “n”) are re‐ported in the figure legends and supporting information figure legends.

## Conflict of Interest

The authors declare no conflict of interest.

## Author Contributions

S. Ling, C. Wu, M. Gui, contributed equally to this work. Qingliang Zhao, Yixin Chen, Shuting Ling, and Mengxuan Gui conceived and designed the project. Shuting Ling performed an animal imaging study and data processing. Chongxin Wu. performed recombinant virus construction and characterization. Shuting Ling, Chongxin Wu, and Mengxuan Gui establish cell lines and perform animal models. These three authors contributed equally to this project. Chongxin Wu, Kaiyun Chen, and Yanbo Yang helped to perform recombinant virus construction. Jiwei Xing performed OCT imaging. Fengxian Du wrote custom code for OCT‐angiography data analysis. Jiwei Xing, Mengxuan Gui, and Luyao Yang performed or helped with imaging and its analyses. Zhaokui Jin and Ningshao Xia assisted with project administration. Shuting Ling, Guosong Wang, Yixin Chen, and Qingliang Zhao analyzed the data and co‐wrote the paper. Guosong Wang, Yixin Chen, and Qingliang Zhao supervised the entire project. All the authors have approved the final version.

## Supporting information



Supporting Information

## Data Availability

The data that support the findings of this study are available in the supplementary material of this article.
