# Supplementary material for: Multimodal Imaging Reveals Cerebral Microcirculation Dynamics and Mechanisms in Mouse Central Nervous System After Intranasal Infection With Recombinant Pseudorabies Virus
Source: Adv Sci (Weinh). 2025 Sep 23;12(48):e08124. doi: 10.1002/advs.202508124 (PMC12752574; doi:10.1002/advs.202508124)
Supplement: Supplementary file 1 — Supporting Information [file ADVS-12-e08124-s001.docx]

Supplementary Materials for

**Multimodal Imaging Reveals Cerebral Microcirculation Dynamics and Mechanisms in Mouse Central Nervous System after Intranasal Infection with Recombinant Pseudorabies Virus**

Shuting Ling ^1^**^†^**, Chongxin Wu^1, 2^**^†^**, Mengxuan Gui^1^**^†^**, Kaiyun Chen^1, 2^, Yanbo Yang^1, 2^, Jiwei Xing^1^, Fengxian Du^1^, Wei Liao^1^, Luyao Yang^1^, Zhaokui Jin^3^, Ningshao Xia^1, 2^, Guosong Wang^1, 2, 4*^, Yixin Chen^1, 2*^, Qingliang Zhao^1*^

^1^State Key Laboratory of Vaccines for Infectious Diseases, Xiang An Biomedicine Laboratory, National Innovation Platform for Industry-Education Integration in Vaccine Research, Center for Molecular Imaging and Translational Medicine, School of Public Health, Xiamen University, Xiamen, Fujian, 361102, China.

^2^State Key Laboratory of Molecular Vaccinology and Molecular Diagnostics, National Institute of Diagnostics and Vaccine Development in Infectious Diseases, School of Life Sciences, School of Public Health, Xiamen University, Xiamen, 361102, Fujian Province, China

^3^School of Biomedical Engineering, Guangzhou Medical University, Guangzhou 511436, China.

^4^Department of Experimental Research, Sichuan Clinical Research Center for Cancer, Sichuan Cancer Hospital & Institute, Sichuan Cancer Center, University of Electronic Science and Technology of China, Chengdu 610042, China.

†These authors contributed equally to this work.

*Corresponding author. Email: [zhaoql@xmu.edu.cn](mailto:zhaoql@xmu.edu.cn) (Q. Z.); yxchen2008@xmu.edu.cn (Y. C.); wangguosong2020@xmu.edu.cn (G.W.).

**This PDF file includes:**

Figs. S1 to S4

**
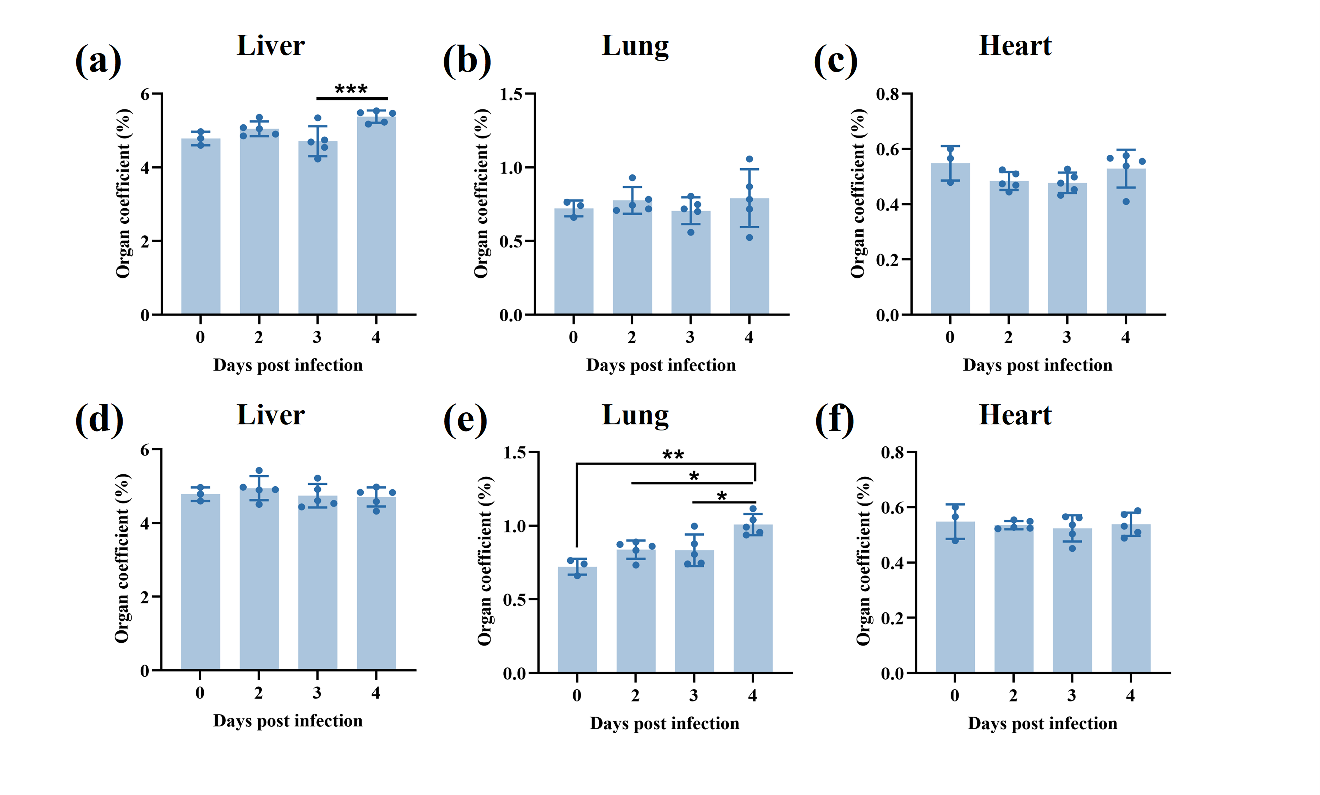
**

**Fig. S1**. **Changes of organ coefficients in mice.** (**a**) Organ coefficients of liver in intraperitoneal infection group (n ≥ 3). The data for day 0 are shared between Figure S1a and Figure S1d. (**b**) Organ coefficients of lung in intraperitoneal infection group (n ≥ 3). The data for day 0 are shared between Figure S1b and Figure S1e. (**c**) Organ coefficients of heart in intraperitoneal infection group (n ≥ 3). The data for day 0 are shared between Figure S1c and Figure S1f. (**d**) Organ coefficients of liver in intranasal infection group (n ≥ 3). (**e**) Organ coefficients of lung in intranasal infection group (n ≥ 3). (**f**) Organ coefficients of heart in intranasal infection group (n ≥ 3). Data shown as mean ± SD, One-way ANOVA in **(a)** - **(f)** followed by Bonferroni *t*-test. * *P*<0.05; ** *P* <001; *** *P* <0.001.


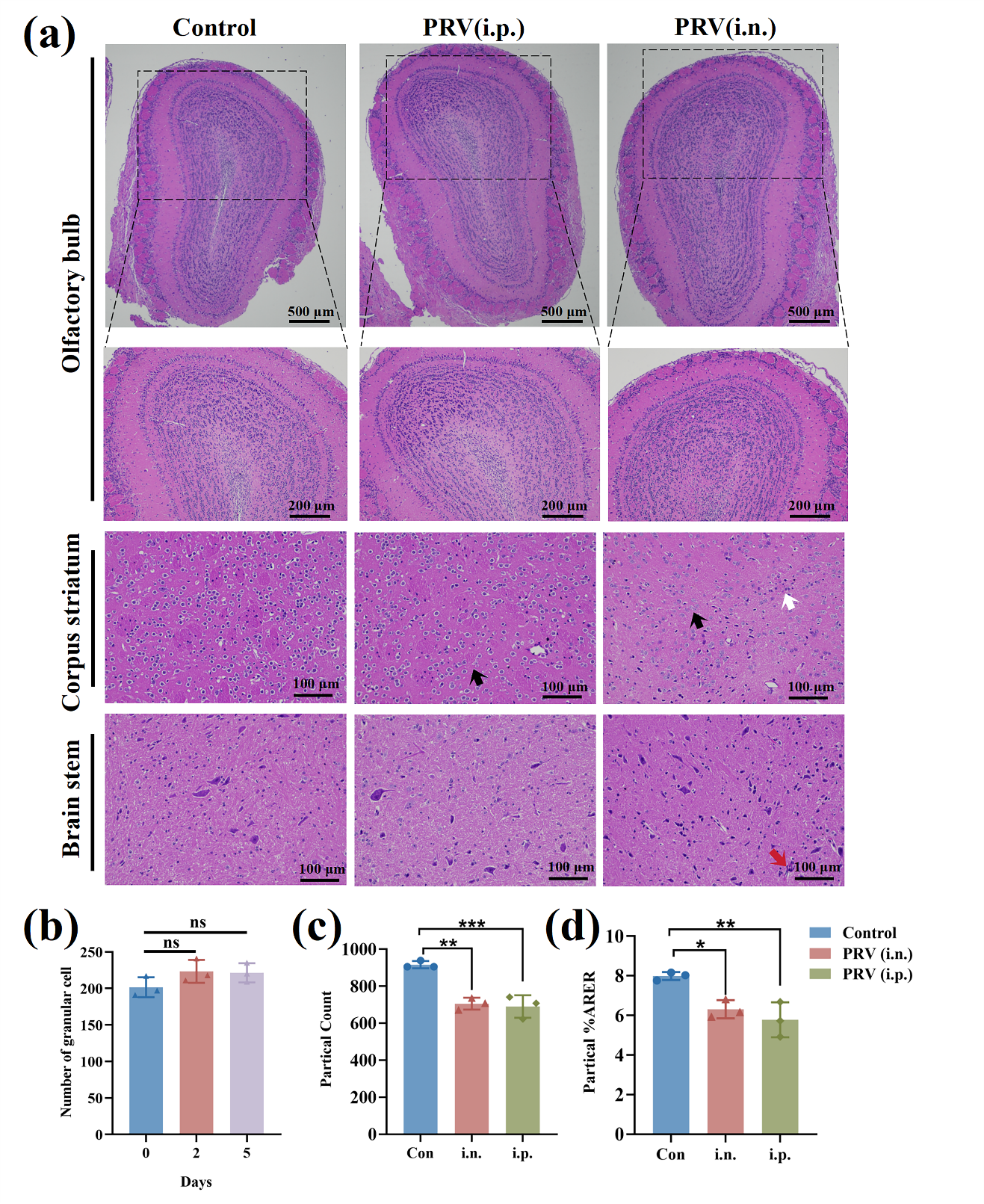


**Fig. S2**. **Histopathological damage to brain tissue.** (**a**) H&E stains image of olfactory bulbs, corpus striatum and brainstem tissue (n = 3). Black arrow: karyolysis; White arrow: tissue necrosis; Red arrow: perivascular inflammatory cell infiltration. (**b**) Number of granular cells in olfactory bulbs (n = 3). (**c**) Number of nuclei in corpus striatum (n = 3). (**d**) Nucleus area ratio in corpus striatum (n = 3). Data shown as mean ± SD, One-way ANOVA in **(b)**, **(c)** and **(d)** followed by Dunn’s post hoc test comparing all groups to the control. ns, no significance; * *P*<0.05; ** *P* <0.01; *** *P* <0.001.

**
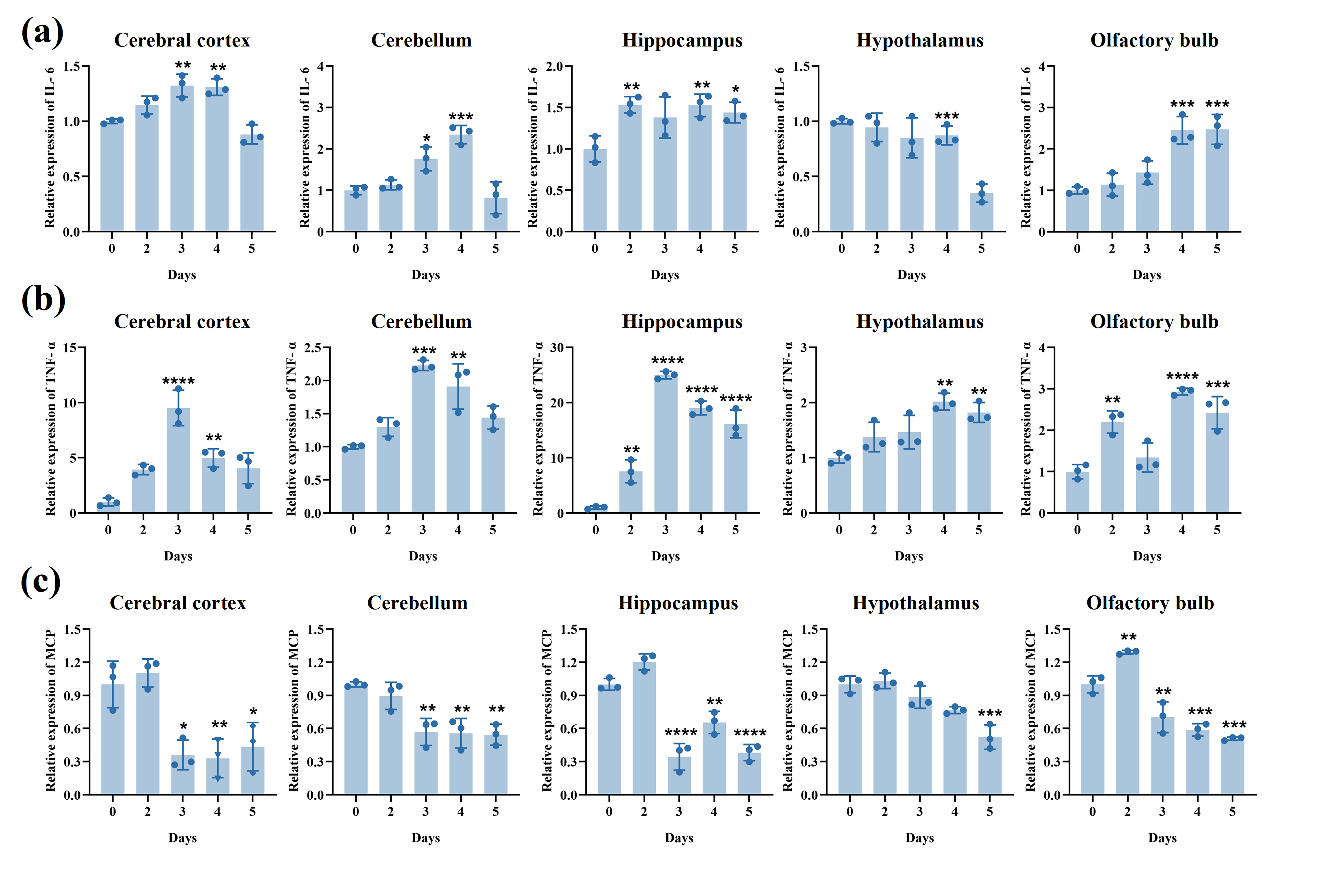
**

**Fig. S3**. **Inflammatory damage of brain tissue in CNS infection.** (**a**) Relative expression of IL-6 in brain of intranasal PRV infected mice (n = 9). (**b**) Relative expression of TNF-α in brain of intranasal PRV infected mice (n = 9). (**c**) Relative expression of MCP-1 in brain of intranasal PRV infected mice (n = 9). Brain tissue from three mice was combined into a single independent sample in Figure S3a-c due to small mass. Data shown as mean ± SD, One-way ANOVA in **(a)**, **(b)**, and **(c)** followed by Dunn’s post hoc test comparing all groups to the day 0. * *P*<0.05; ** *P* <0.01; *** *P* <0.001; **** *P* <0.0001.

**
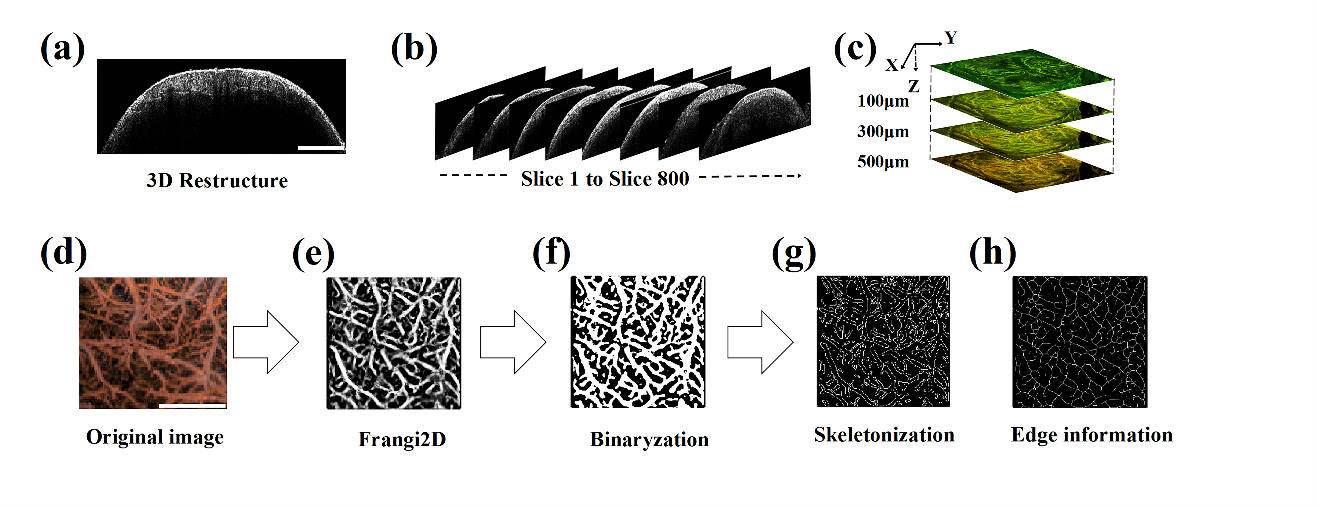
**

**Fig. S4**. **Analysis of OCTA images.** (**a**) B-scan images of mouse brain. (**b**) Schematic representation of 3D brain structure reconstruction. (**c**) OCTA color-coded images of brain at different depths. (**d**) OCTA color-coded map of primary visual cortex in mice. (**e**) Vascular reconstruction map based on Hessian matrix. (**f**) Binary map of vascular regions. (**g**) Vascular skeleton diagram. (**h**) Information map of vessel margins. Scale bar: 200 μm.
